# Frailty and Mortality Risk in COPD: A Cohort Study Comparing the Fried Frailty Phenotype and Short Physical Performance Battery

**DOI:** 10.2147/COPD.S375142

**Published:** 2023-01-20

**Authors:** Lisa Jane Brighton, Claire M Nolan, Ruth E Barker, Suhani Patel, Jessica A Walsh, Oliver Polgar, Samantha S C Kon, Wei Gao, Catherine J Evans, Matthew Maddocks, William D C Man

**Affiliations:** 1 Cicely Saunders Institute of Palliative Care, Policy and Rehabilitation, King’s College London, London, UK; 2 Harefield Respiratory Research Group, Royal Brompton and Harefield Hospitals, Guy’s and St Thomas NHS Foundation Trust, London, UK; 3 Division of Physiotherapy, College of Health, Medicine and Life Sciences, Brunel University London, London, UK; 4 National Heart and Lung Institute, Imperial College, London, UK; 5 Insight Innovation, Wessex Academic Health Science Network, Southampton, UK; 6 Department of Respiratory Medicine, The Hillingdon Hospital NHS Trust, London, UK; 7 Brighton General Hospital, Sussex Community NHS Foundation Trust, Brighton, UK; 8 Harefield Pulmonary Rehabilitation Unit, Guy’s and St Thomas NHS Foundation Trust, London, UK; 9 Faculty of Life Sciences & Medicine, King’s College London, London, UK

**Keywords:** respiratory disease, chronic obstructive pulmonary disease, frailty, survival

## Abstract

**Background:**

Identifying frailty in people with chronic obstructive pulmonary disease (COPD) is deemed important, yet comparative characteristics of the most commonly used frailty measures in COPD are unknown. This study aimed to compare how the Fried Frailty Phenotype (FFP) and Short Physical Performance Battery (SPPB) characterise frailty in people with stable COPD, including prevalence of and overlap in identification of frailty, disease and health characteristics of those identified as living with frailty, and predictive value in relation to survival time.

**Methods:**

Cohort study of people with stable COPD attending outpatient clinics. Agreement between frailty classifications was described using Cohen’s Kappa. Disease and health characteristics of frail versus not frail participants were compared using t-, Mann–Whitney U and Chi-Square tests. Predictive value for mortality was examined with multivariable Cox regression.

**Results:**

Of 714 participants, 421 (59%) were male, mean age 69.9 years (SD 9.7), mean survival time 2270 days (95% CI 2185–2355). Similar proportions were identified as frail using the FFP (26.2%) and SPPB (23.7%) measures; classifications as frail or not frail matched in 572 (80.1%) cases, showing moderate agreement (Kappa = 0.469, SE = 0.038, p < 0.001). Discrepancies seemed driven by FFP exhaustion and weight loss criteria and the SPPB balance component. People with frailty by either measure had worse exercise capacity, health-related quality of life, breathlessness, depression and dependence in activities of daily living. In multivariable analysis controlling for the Age Dyspnoea Obstruction index, sex, BMI, comorbidities and exercise capacity, both the FFP and SPPB had predictive value in relation to mortality (FFP aHR = 1.31 [95% CI 1.03–1.66]; SPPB aHR = 1.29 [95% CI 0.99–1.68]).

**Conclusion:**

In stable COPD, both the FFP and SPPB identify similar proportions of people living with/without frailty, the majority with matching classifications. Both measures can identify individuals with multidimensional health challenges and increased mortality risk and provide additional information alongside established prognostic variables.

## Introduction

Approximately one in five people with COPD are also living with frailty.^1^ Frailty is a multidimensional syndrome, characterised by decreased reserve and diminished resistance to stressors.^2^ It is relevant across diagnoses, including multimorbidity, and can provide a holistic measure of a person’s health and risk of adverse outcomes. People with both COPD and frailty experience poorer physical and mental health,^3^ higher risk of readmission^4^ and mortality,^5^ and are at higher risk of not receiving disease modifying treatments^3^,^6^ compared to those with COPD without frailty. Identifying frailty in respiratory research and practice has been recognised as important by public and professional stakeholders.^7^

Several measures have been used to identify frailty in people with COPD, and there is no universal agreement on which frailty measure should be used.^8^ While comprehensive geriatric assessment is the gold-standard approach to identify this syndrome and direct appropriate clinical care,^9^ brief tools to approximate frailty are essential to identify potential candidates for additional support, and measure frailty as a clinical or research outcome. The Fried Frailty Phenotype (FFP) is one of the most well-established measures of frailty,^8^ comprising five characteristics: unintentional weight loss, exhaustion, low physical activity, slowness and weakness.^10^ The Short Physical Performance Battery (SPPB)^11^ incorporates static balance tests, four-metre gait speed (4MGS), and the five sit-to-stand test, and has recently been recommended by the European Medicines Agency for baseline characterisation of physical frailty in people aged ≥65 years enrolled in clinical trials. Both measures are responsive to change following pulmonary rehabilitation^3^,^12^ and predictive of adverse events,^13^,^14^ including mortality.^14^ Using the FFP, people with COPD and frailty have been found to have higher risk of mortality compared to people with COPD without frailty (adjusted HR 1.4; 95% CI 0.97 to 2.0);^15^ and compared to people with neither COPD nor frailty (adjusted HR 2.7, 95% CI 1.07–6.94).^16^ While SPPB scores are predictive of mortality in COPD,^14^ this has not been explored with SPPB scores dichotomised by thresholds for frail versus not frail.

Although both the FFP and SPPB measures have been used to identify people living with frailty, little is known about the comparative characteristics of these measures when used with people with COPD. One study with 395 lung transplant candidates measured frailty using both measures to assess their construct and predictive validity.^6^ Despite more people being categorised as frail using FFP versus SPPB (28% vs 10%), both measures were associated with physiological and functional baseline characteristics and outcomes. However, only 30% of participants had COPD, and this study did not explore associations between the frailty measures and broader domains of health (eg, psychological, quality of life). Moreover, the multivariate modelling did not control for any widely used and validated prognostic index (eg, Age Dyspnoea Obstruction [ADO] or Body mass index, Obstruction, Dyspnoea, Exercise performance [BODE]).^17^

How the FFP and SPPB identify people living with frailty, and their varying predictive properties, may have important implications for their use and interpretation. Yet, these measures have not been directly compared in people living with COPD. Differences in the frailty definitions selected may modify the target population and interventional response and/or inform how evidence relating to frailty is synthesised. To support data-driven decision-making in clinical practice and research, this study aimed to compare the FFP and SPPB measures of frailty in people with stable COPD. Objectives were to (a) describe prevalence of, and overlap in, identification of frailty using the two measures; (b) compare disease and health characteristics in those identified as living with frailty using the two measures, and (c) compare the predictive value of the two frailty measures in relation to survival time.

## Methods

### Design

Cohort study.

### Setting

Hillingdon Borough, North West London, United Kingdom.

### Participants

Participants were consecutively identified and recruited from community respiratory and pulmonary rehabilitation assessment clinics, between November 2011 and January 2015. Eligible participants included people aged 35 years or over with a physician diagnosis of COPD (consistent with GOLD criteria^18^), and appropriate for pulmonary rehabilitation referral in line with British Thoracic Society Guidance: able to walk at least five metres, experiencing functional impairment due to breathlessness, and no previous supervised pulmonary rehabilitation in the previous 12 months. Exclusions included exacerbation of their COPD within the past four weeks that required a change in medication, or if moderate-intensity exercise was deemed unsafe (eg, due to unstable cardiac condition). Data from this ongoing research cohort have been published previously.^3^,^19^ The current study includes those with complete data for both frailty measures. Where people were assessed for pulmonary rehabilitation more than once during the study period, only their first assessment was included.

### Frailty Measures

We compared the FFP and the SPPB, collected at baseline assessments.

The five characteristics of the FFP were assessed, respectively, using self-report unintentional weight loss history, two self-report questions on exhaustion from the Centre for Epidemiological Studies Depression (CES-D) questionnaire, self-reported physical activity from the modified Minnesota Leisure-Time physical activity questionnaire, handgrip dynamometry (weakness), and 4MGS (slowness). The 4MGS was completed using processes validated in COPD^20^ on a flat, unobstructed course, following a demonstration by the assessor. Participants were able to use their usual walking aids if applicable, and the faster of two attempts completed sequentially without rest was used. Presence or absence of each FFP characteristic was assessed and scored based on standardised criteria, described in detail previously.^3^ People meeting three or more criteria were considered to be living with frailty;^10^ those meeting 1–2 (prefrail) or 0 criteria (robust) were considered not to be living with frailty.

For the SPPB,^11^,^21^ performance in static balance, 4MGS, and five sit-to-stand tests were each assessed following a standardised protocol from the National Institute of Ageing, and scored from 0 to 4. The sit-to-stand component followed processes validated in COPD,^22^ including the use of a straight-backed armless chair with a floor-to-seat height of 48cm. Participants began with an initial stand and sit: those completing this successfully completed the five sit-to-stands, while the test was terminated for those unable to complete this initial manoeuvre. Each SPPB component contributes to a total score from 0 to 12, with higher scores indicating robustness. People scoring ≤7 were considered to be living with frailty,^21^ in line with European Medicines Agency guidance. As there is no consensus over optimal cut-offs when using the SPPB, we also conducted sensitivity analyses using alternative cut-off values of ≤8^23^ and ≤9.^24^

### Analysis

#### Prevalence and Overlap in Identification of Frailty

The prevalence of participants identified as living with frailty using each measure were described as percentages, and agreement described using Cohen’s Kappa. Agreement was categorised: slight ≤0.20, fair 0.21–0.40, moderate 0.41–0.60, substantial 0.61–0.80, almost perfect 0.81–1.00.^25^ Overlap in frailty categorisation between the two measures was illustrated using a Venn diagram. Post-hoc analysis explored areas of convergence and divergence between the measures through tabulating and examining inter-item correlations.

#### Comparison of Population Characteristics

The following characteristics (scale, ranges if applicable) from participants’ baseline assessment were described for those identified as living with or without frailty by each measure: age (years); forced expiratory volume in one second percent-predicted (FEV_1_% predicted); breathlessness (Medical Research Council [MRC] Dyspnoea, 1–5); Age Dyspnoea Obstruction (ADO) Index (0–14); Body Mass Index (BMI); comorbidities (age-adjusted Charlson comorbidity index); exercise capacity (Incremental Shuttle Walk Test [ISWT] distance in metres); anxiety symptoms (Hospital Anxiety and Depression Scale [HADS], 0–21); depression symptoms (HADS, 0–21); health-related quality of life (Chronic Respiratory Questionnaire Dyspnoea [5–35], Emotion [7–49], Fatigue [4–28] and Mastery [4–28] domains); and independence in basic activities of daily living (Katz questionnaire, scores 1–6 dichotomised some dependence [scores 1–5] and independent [score 6]). Questionnaires and physical measures were collected during their assessment in an outpatient consultation room. Additional information about these measures can be found within the Supplementary Material Table S1.

Following distribution checks for normality, characteristics were described using mean/medians and standard deviations/interquartile ranges (as appropriate) for continuous variables, and using frequencies and percentages for categorical variables. Independent *t*-tests/Mann Whitney *U*-tests and chi squared tests (as appropriate) were used to compare those identified as living with and not living with frailty within each measure. A p-value of less than 0.01 was used as the threshold for statistical significance to reduce risk of type 1 error due to multiple testing.^26^

#### Predictive Value for Mortality

It is recommended that, in survival analysis, there should be a minimum of 10 events per independent variable included in the model.^27^ As there were 376 deaths, there were sufficient cases for multivariable modelling.

Participants were followed up prospectively, and date of death was identified from hospital records and/or central National Health Service databases. Time to death in days was calculated from the date of assessment until date of death. Participants who survived were censored on 29th January 2021.

Kaplan–Meier plots and log rank tests were used to assess whether each frailty measure identified groups with different survival curves. The following disease and health characteristics were also assessed for associations with mortality using univariate Cox regression (or appropriate alternatives if proportional hazard assumption was violated), to inform subsequent adjusted analysis: Body Mass Index, comorbidity index, exercise capacity, anxiety, depression, independence in activities of daily living, and pulmonary rehabilitation completion. In separate models for each frailty measure, variables associated with mortality in univariable analyses (p < 0.05) were included in multivariable Cox Regression analysis (or appropriate alternatives if proportional hazard assumption was violated). In all cases, the multivariable analyses included checking for collinearity (r < 0.75), and controlling for sex and the ADO index: the former to account for known sex differences in mortality,^28^ the latter to determine the prognostic value of the FFP and SPPB over and above an established validated prognostic indicator.^29^ Analyses were undertaken using IBM SPSS Statistics 27.^30^

### Ethical Approval

Study procedures complied with the Declaration of Helsinki. All participants gave informed consent. The recruitment and follow-up of the cohort received ethical approval from the West London (11/H0707/2) and London Camberwell St Giles (11/LO/1780) research ethics committees.

## Results

### Participant Characteristics

Of 1084 unique referrals for people with COPD during the study period, 1019 attended their assessment. Of these, 716 (70%) were eligible to be included in the research cohort. Of 716 individual participant assessments during the study period, SPPB scores were missing for 2 participants and the remaining 714 had data for both frailty measures. Four-hundred and twenty-one (59%) were male, and the mean (SD) age was 69.9 (9.7) years. Participant characteristics are shown in Table 1.

**Table 1:** Participant Characteristics (n = 714)

	Median [IQR], Mean (SD) or n (%)
Male	421 (59%)
Age	69.9 (9.7)
Medical Research Council Dyspnoea	3 [2–4]
FEV_1_% predicted^†^	47 [33–63]
ADO score^†^	9.3 (2.3)
BMI	27 [23.1–31.8]
Age-adjusted Charlson Comorbidity Index	4 [3–5]
Smoking status	
Current	124 (17.4%)
Former	540 (75.6%)
Never	47 (6.6%)
Missing	3 (0.4%)
Oxygen therapy	
Long-term	35 (4.9%)
Ambulatory	48 (6.7%)
Incremental Shuttle Walk Test (metres)	200 [100–310]
Hospital Anxiety and Depression Scale	
Anxiety symptoms*	7 [3–11]
Depression symptoms*	6 [4–9]
Chronic Respiratory Questionnaire	
Dyspnoea	13 [10–17]
Fatigue	13 [9–17]
Emotion	30 [23–38]
Mastery	18 [13–22]
Katz questionnaire	
Some dependence in Activities of Daily Living	164 (23.0%)
Independent in Activities of Daily Living	548 (76.8%)
Missing	2 (0.03%)

**Notes**: Data are median [IQR] or n (%) except age and ADO reported as mean (SD). *Higher scores indicate poorer function, ^†^n = 712.

**Abbreviations**: ADO, Age Dyspnoea Obstruction Index; BMI, body mass index; FEV_1_, Forced Expiratory Volume in 1 second; IQR, inter-quartile range; SD, standard deviation.

### Prevalence and Overlap in Frailty Identification

**Figure 1:**
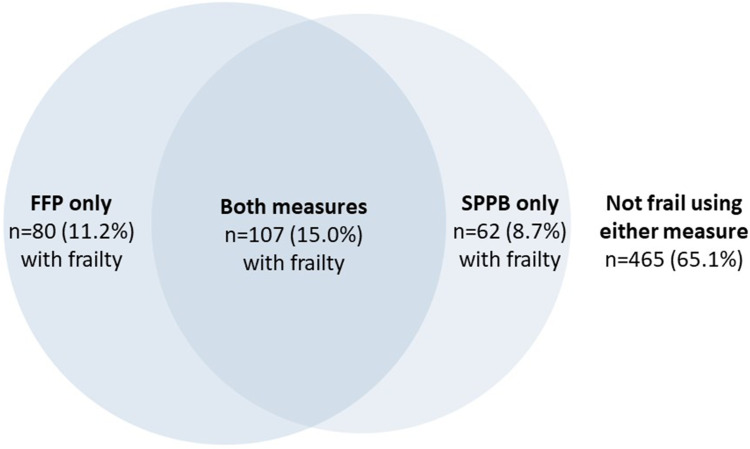
Venn diagram of frailty classification using Fried Frailty Phenotype (FFP) and Short Physical Performance Battery (SPPB) measures (n = 714).

Similar proportions of the sample were identified as living with frailty using the FFP (26.2%, n = 187) and SPPB (23.7%, n = 169) measure. There was moderate agreement between the measures (K = 0.469, SE = 0.038, p = <0.001), with matching classifications of frail or not frail in 572 (80.1%) of cases (Figure 1). Sensitivity analysis using SPPB cut-offs of ≤8 and ≤9 led to higher proportions of the sample being identified as frail (33.6% [n = 240] and 46.1% [n = 329], respectively), but lower proportions of matching classifications (76.9% [n = 549] and 70.0% [n = 500], respectively) and lower kappa agreement scores with the FFP (0.452 and 0.377, respectively).

Post-hoc analyses of inter-item correlations (Table 2) suggest that classification discrepancies may have arisen particularly from the weight loss and exhaustion components of the FFP, both of which show the lowest correlations with each SPPB item. Balance was the SPPB item least correlated with the FFP items.

**Table 2:** Inter-Item Correlation Between Fried Frailty Phenotype and Short Physical Performance Battery Components

		Fried Frailty Phenotype				
		Weight Loss	Exhaustion	Low Physical Activity	Slowness	Weakness
Short Physical Performance Battery	Balance	−0.001	0.120	0.169	0.334	0.215
	Gait speed	0.048	0.165	0.328	0.839	0.242
	Sit to stand	0.099	0.151	0.245	0.501	0.284

### Disease and Health Characteristics by Frailty Measure

Participants identified as living with frailty using either the FFP or SPPB were significantly older and had more comorbid conditions but did not show substantial differences in FEV_1_% predicted or BMI (Table 3). Participants with frailty identified using either measure scored lower on functional exercise capacity and reported more breathlessness and dependence in activities of daily living, higher depression symptoms, and poorer quality of life on the CRQ domains of fatigue, emotion, and mastery. Only participants identified as living with frailty using the FFP (not SPPB) reported significantly poorer anxiety and worse CRQ dyspnoea. Sensitivity analysis using cut-offs of ≤8 and ≤9 for SPPB found similar patterns, but as the cut-off score increased the SPPB showed significant differences in anxiety (≤8 only) and CRQ dyspnoea (≤8 and ≤9) between those with and without frailty.

**Table 3:** Comparison of People Identified as Living with Frailty versus without Frailty Using the Fried Frailty Phenotype and Short Physical Performance Battery (n = 714)

	Fried Frailty Phenotype			Short Physical Performance Battery		
	Not Frail (n = 527)	Frail (n = 187)	*P* value	Not Frail (n = 545)	Frail (n = 169)	*P* value
	Median [IQR], Mean (SD) or n (%)	Median [IQR], Mean (SD) or n (%)		Median [IQR], Mean (SD) or n (%)	Median [IQR], Mean (SD) or n (%)	
Age	68.9 (9.4)	72.6 (10.1)	<0.001*	68.5 (9.5)	74.2 (9.3)	<0.001*
Medical Research Council Dyspnoea	3 [2–4]	4 [4–5]	<0.001*	3 [2–4]	4 [3–5]	<0.001*
FEV_1_% predicted^†^	48 [34.0–63.3]	44 [30.0–59.0]	0.028	47 [33.3–63.0]	46 [31.0–62.0]	0.532
ADO score^†^	8.88 (2.3)	10.34 (2.0)	<0.001*	8.91 (2.29)	10.38 (1.98)	<0.001*
BMI (kg/m^2^)	26.9 [23.2–31.7]	27.4 [22.4–32.4]	0.918	26.9 [23.0–31.8]	27.4 [23.2–32.3]	0.400
Age-adjusted Charlson Comorbidity Index	4 [3–5]	5 [4–6]	<0.001*	4 [3–5]	5 [4–6]	<0.001*
Incremental Shuttle Walk Test (metres)	250 [150–340]	80 [40–160]	<0.001*	250 [150–340]	70 [30–120]	<0.001*
Hospital Anxiety and Depression Scale						
Anxiety symptoms^‡^	6 [3–10]	8 [4–13]	<0.001*	6 [3–10]	7 [4–12]	0.100
Depression symptoms^‡^	6 [3–8]	8 [5–11]	<0.001*	6 [3–9]	7 [5–10]	<0.001*
Chronic Respiratory Questionnaire						
Dyspnoea	13 [10–17]	12 [9–15]	0.001*	13 [10–17]	13 [10–16]	0.474
Fatigue	14 [10–18]	10 [8–13]	<0.001*	14 [10–18]	11 [8–14]	<0.001*
Emotion	32 [26–39]	26 [19–32]	<0.001*	31 [24–39]	28 [22–34]	0.001*
Mastery	18 [14–23]	14 [11–19]	<0.001*	18 [13–23]	16 [12–21]	0.001*
Katz questionnaire						
Some dependence in Activities of Daily Living	101 (19.2%)	63 (33.9%)	<0.001*	108 (19.9%)	56 (33.1%)	0.001*
Independent in Activities of Daily Living	425 (80.8%)	123 (66.1%)		435 (80.1%)	113 (66.9%)	

**Notes**: Data are median [IQR] or n (%) except age and ADO reported as mean (SD). All tests were non-parametric except for age and ADO (which were normally distributed); *Significant at p<0.010; ^†^n = 712; ^‡^Higher scores indicate poorer function.

**Abbreviations**: FEV_1_, forced expiratory volume in 1 second; ADO, Age Dyspnoea Obstruction Index; BMI, body mass index; IQR, inter-quartile range; SD, standard deviation.

### Predictive Value in Relation to Survival

Of the 714 participants, 376 (52.7%) had died by 29th January 2021. Mean survival time was 2270 days (95% CI 2185–2355); approximately 6 years. For both the FFP and SPPB measure, a higher proportion of people with frailty had died by end of the study period than the non-frail groups: FFP 71.7% (n = 134) with frailty vs 45.9% (n = 242) without frailty died; SPPB 72.2% (n = 122) with frailty vs 46.6% (n = 254) without frailty died.

**Figure 2:**
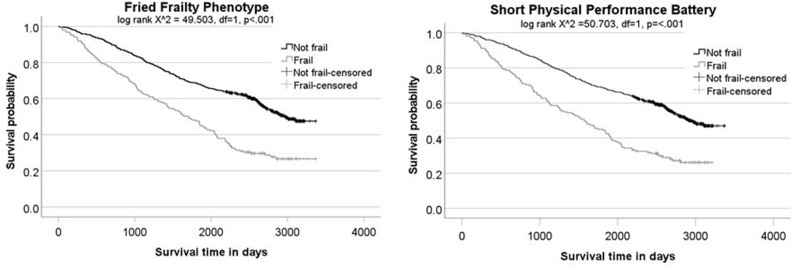
Kaplan–Meier plots showing survival of frail vs non-frail groups using the Fried Frailty Phenotype and Short Physical Performance Battery.

Survival time was approximately 2 years shorter for those with frailty versus without frailty, using either the FFP (mean 1795 days [95% CI 1629–1961] vs mean 2439 days [95% CI 2344–2533]) or SPPB (mean 1698 days [95% CI 1530–1866] vs 2435 days [95% CI 2342–2527]). As illustrated in the Kaplan–Meier plots in Figure 2, both measures identified a frail group with significantly shorter survival than the group who were not frail.

Univariate Cox regression analysis found that BMI, comorbidities, and exercise capacity were also significantly related to survival, while activities of daily living, anxiety, depression, and pulmonary rehabilitation completion were not. The final multivariable models for each frailty measure and survival included ADO and sex (as forced variables) as well as comorbidities, exercise capacity and BMI. When controlling for these variables, frailty measured using the FFP measure remained a significant independent predictor of survival, while frailty measured using the SPPB did not. However, both showed comparable point estimates, suggesting in either case an increase in mortality risk for those with frailty (Table 4). Sensitivity analysis using the alternative SPPB cut-offs of ≤8 and ≤9 found similar results (≤8 cut-off HR = 1.73, 95% CI 1.41–2.12 and aHR = 1.00, 95% CI 0.77–1.29; ≤9 cut-off HR = 1.78, 95% CI 1.45–2.18 and aHR = 1.04, 95% CI 0.81–1.33).

**Table 4:** Univariable and Multivariable Prediction of Mortality Comparing the Fried Frailty Phenotype and Short Physical Performance Battery

	Univariable Analysis (n = 714)		Multivariable Analysis:Fried Frailty Phenotype (n = 712)		Multivariable Analysis: SPPB (n = 712)	
	HR (95% CI)	*p*-value	aHR (95% CI)	*p*-value	aHR (95% CI)	*p*-value
Fried Frailty Phenotype	2.10 (1.70–2.60)	<0.001	1.31 (1.03–1.66)	0.031		
Short Physical Performance Battery	2.16 (1.74–2.68)	<0.001			1.29 (0.99–1.68)	0.060
ADO score^‡^	1.28 (1.22–1.35)	<0.001	1.12 (1.05–1.18)	<0.001	1.12 (1.05–1.18)	<0.001
Male Sex*	1.25 (1.01–1.54)	0.038	1.43 (1.16–1.77)	0.001	1.43 (1.16–1.77)	0.001
BMI (kg/m^2^)	0.95 (0.94–0.97)	<0.001	0.95 (0.93–0.96)	<0.001	0.95 (0.93–0.96)	<0.001
Age-adjusted Charlson Comorbidity Index	1.29 (1.22–1.37)	<0.001	1.16 (1.08–1.24)	<0.001	1.16 (1.08–1.24)	<0.001
Incremental Shuttle Walk Test (meters)	0.997 (0.996–0.998)	<0.001	0.998 (0.997–0.999)	<0.001	0.998 (0.997–0.999)	<0.001
Hospital Anxiety and Depression Scale						
Anxiety symptoms	0.99 (0.97–1.02)	0.541				
Depression symptoms	1.02 (1.00–1.05)	0.081				
Some dependence in Activities of Daily Living^†^	1.05 (0.83–1.34)	0.669				
Pulmonary Rehabilitation Completion	0.81 (0.65–1.01)	0.062				

**Notes**: *Reference: Female; ^†^Reference: ADL independence, ^‡^n = 712. Fried Frailty Phenotype model details: −2 log likelihood = 4432.91 X^2^= 188.7, df = 6, p<0.001; Short Physical Performance Battery model details: −2 log likelihood = 4434.00; X^2^= 188.18, df = 6, p<0.001.

**Abbreviations**: ADO, Age Dyspnoea Obstruction Index; BMI, body mass index; HR, hazard ratio; aHR, adjusted hazard ratio; CI, confidence interval.

## Discussion

This study compared the properties of the FFP and SPPB measures in people with COPD. We found moderate agreement in frailty classification, including matching classification of frail or not frail in 80% cases. Participants identified as living with frailty using either measure differed significantly from non-frail participants in similar ways: they were older, had more comorbidities and lower functional exercise capacity, and reported more dependence in activities of daily living, higher depression symptoms, and poorer health-related quality of life. People identified as frail using the FFP also reported significantly worse anxiety symptoms. Both measures showed predictive value in relation to survival. While the FFP provided slightly higher independent predictive value than the SPPB when used alongside other measures, including the ADO Index, this difference was marginal and trivial.

This study is the largest to date to use either the validated version of the FFP measure or the SPPB to predict mortality in people with COPD. Building on prior work by Singer et al that compared these measures in 395 candidates for lung transplant,^6^ we also found approximately 80% matching classifications between the two measures. Moreover, our adjusted hazard ratios for mortality were similar to those for delisting or death before lung transplant (FFP aHR 1.30, 95% CI 1.01–1.67; SPPB aHR 1.53, 95% CI 1.19–1.59).^6^ Together with smaller studies of the FFP^13^,^16^ and SPPB^14^ measures in people with COPD, there is growing evidence that each measure provides additional prognostic information when predicting mortality in this population, even when including established indexes such as ADO, in the current study, and BODE in the study by Fermont et al^14^

The FFP and SPPB both identified a group with multidimensional health challenges. Corroborating previous work, we found that around 1 in 4 people with COPD attending pulmonary rehabilitation were living with frailty^31^,^32^ and that those with frailty on either measure had lower exercise capacity,^6^,^12^ poorer physical function^33^,^34^ and increased breathlessness,^12^,^13^,^33^,^34^ but little difference in lung function.^6^,^12^,^33^ We extend these findings by illustrating associations of frailty, on either measure, with other dimensions of health, including higher depression symptoms, increased dependence in activities of daily living, and lower health-related quality of life. These differences tended to not only be significant but clinically meaningful.^35^,^36^ These wider correlates of frailty are in line with qualitative descriptions of the multidimensional losses experienced by people living with both COPD and frailty.^37^ The FFP measure additionally discriminated between people with different levels of anxiety and CRQ dyspnoea where the SPPB did not. This may reflect closer links between these broader self-reported aspects of health and the self-reported components of the FFP, such as exhaustion.

Measurement of frailty in respiratory research and care is increasingly recognised as important.^7^,^38^ Given varying resources and equipment available across settings (eg, handgrip dynamometers), it is helpful to know that there is substantial overlap between those identified as frail using the FFP or SPPB measure and that both measures identify people experiencing multidimensional health challenges. Decisions driven by pragmatic considerations can now be made with an understanding of the different emphases of each measure. For example, the FFP may identify people with more psychological symptoms and be less discriminant in relation to the presence of balance difficulties, while the SPPB may be less discriminant in relation to presence of exhaustion and weight loss. Moreover, this knowledge may inform more purposive use of either measure, for example, depending on the theorised mechanisms and targets of a particular intervention. Importantly, it should be acknowledged that both the FFP and SPPB are only surrogate markers of frailty: a comprehensive geriatric assessment remains the gold-standard approach to identify this syndrome and direct appropriate clinical care.^9^

Our data show that those identified as frail using the FFP or SPPB are twice as likely to die in the subsequent six years or so than their non-frail counterparts. Although there are limited trial data, growing evidence supports the potential of pulmonary rehabilitation in reversing frailty,^3^,^32^ but also of the difficulties those with frailty face in completing this intervention.^3^,^37^ Adapted pulmonary rehabilitation approaches for this group that integrate comprehensive geriatric assessment may have a role here,^39^ and work in this area is ongoing.^40^ Alongside this, the increased risk of mortality and poorer multidimensional health in those with COPD and frailty should also prompt thinking around the information and support needs of this group, which might include a role for integrated working with palliative care specialists and advance care planning.^41^

Although the single centre design and restriction to people attending an initial pulmonary rehabilitation assessment may reduce external validity, the large sample size and consecutive recruitment may support some generalisability to other outpatient cohorts. The focus on baseline data (with only survival as follow-up data) also meant little frailty data was missing for this cohort. This analysis included relevant disease characteristics, physical tests and self-reported health across multiple dimensions, including physical and psychological symptoms, activities of daily living and quality of life. This allowed us to comprehensively characterise those with frailty, but also adjust for several important confounders. These measures are routinely collected by skilled professionals during clinical assessments, supporting internal validity. It is important to acknowledge that including the separate component variables for Age, Dyspnoea and Obstruction may have accounted for more variance in the multivariate modelling than the composite ADO index, however it was deemed valuable to understand the prognostic value of the FFP and SPPB over and above an established prognostic indicator. Our long-term mortality follow-up helps demonstrate the value of two common frailty measures over an extended duration, but future work exploring comparative predictive value in relation to hospitalisation and readmission may also be useful. Importantly, this comparison only included two measures of frailty, both of which require physical tests which are not always feasible or practical. Further comparative work exploring the properties of other types of frailty measure including self-report screening tools (eg, FRAIL Scale^42^) and clinical-judgement-based approaches (eg, the Clinical Frailty Scale^43^) in COPD is needed. In addition, applicability across different ethnicities is unknown due to lack of data on this characteristic.

In conclusion, we found that in stable COPD, both the FFP and SPPB measures identify people with multidimensional health challenges and increased mortality risk. When used alongside other established measures, including the ADO index, both the FFP and SPPB frailty measures offer added value in predicting mortality.

## Data Sharing Statement

All data requests should be submitted to Dr William D-C Man (w.man@rbht.nhs.uk) for consideration. Access to anonymised data might be granted following investigator review.
